# Effects of Frontal Theta Rhythms in a Prior Resting State on the Subsequent Motor Imagery Brain-Computer Interface Performance

**DOI:** 10.3389/fnins.2021.663101

**Published:** 2021-08-13

**Authors:** Jae-Hwan Kang, Joosang Youn, Sung-Hee Kim, Junsuk Kim

**Affiliations:** ^1^AI Grand ICT Research Center, Dong-eui University, Busan, South Korea; ^2^Department of Industrial ICT Engineering, Dong-eui University, Busan, South Korea

**Keywords:** electroencephalography, brain computer interface, recurrent quantification analysis, BCI illiteracy, convolution neural network

## Abstract

Dealing with subjects who are unable to attain a proper level of performance, that is, those with brain–computer interface (BCI) illiteracy or BCI inefficients, is still a major issue in human electroencephalography (EEG) BCI systems. The most suitable approach to address this issue is to analyze the EEG signals of individual subjects independently recorded before the main BCI tasks, to evaluate their performance on these tasks. This study mainly focused on non-linear analyses and deep learning techniques to investigate the significant relationship between the intrinsic characteristics of a prior idle resting state and the subsequent BCI performance. To achieve this main objective, a public EEG motor/movement imagery dataset that constituted two individual EEG signals recorded from an idle resting state and a motor imagery BCI task was used in this study. For the EEG processing in the prior resting state, spectral analysis but also non-linear analyses, such as sample entropy, permutation entropy, and recurrent quantification analyses (RQA), were performed to obtain individual groups of EEG features to represent intrinsic EEG characteristics in the subject. For the EEG signals in the BCI tasks, four individual decoding methods, as a filter-bank common spatial pattern-based classifier and three types of convolution neural network-based classifiers, quantified the subsequent BCI performance in the subject. Statistical linear regression and ANOVA with *post hoc* analyses verified the significant relationship between non-linear EEG features in the prior resting state and three types of BCI performance as low-, intermediate-, and high-performance groups that were statistically discriminated by the subsequent BCI performance. As a result, we found that the frontal theta rhythm ranging from 4 to 8 Hz during the eyes open condition was highly associated with the subsequent BCI performance. The RQA findings that higher determinism and lower mean recurrent time were mainly observed in higher-performance groups indicate that more regular and stable properties in the EEG signals over the frontal regions during the prior resting state would provide a critical clue to assess an individual BCI ability in the following motor imagery task.

## Introduction

As a means of controlling external devices without real limb movement, motor imagery (MI)-based brain-computer interface (BCI) technology enables the translation of the user’s motor intentions into specific commands to perform the corresponding actions (Schalk et al., 2004; Lotte et al., 2018). MI-BCI technology has been widely used not only in the neurorehabilitation system for subjects to recover their sensorimotor ability after stroke (Ang et al., 2010; Leamy et al., 2014), but also as a ubiquitous system for healthy individuals to control external devices (Liao et al., 2012; Marshall et al., 2013; He et al., 2015; Kim et al., 2019). Non-invasively, scalp electroencephalography (EEG) signals, which record electrical activity in the brain from the scalp surface, have been regarded as the most important modality for the MI-BCI system that can be decoded by a variety of signal processing and decoding methods (Nicolas-Alonso and Gomez-Gil, 2012; Padfield et al., 2019; Stegman et al., 2020). Although there have been many advances in the field of EEG-BCI technology over the decades, BCI illiteracy still remains a major problem (Vidaurre and Blankertz, 2010). Although the inappropriate usage of this terminology is controversial (Thompson, 2019), some specific terms such as BCI-illiteracy, BCI-deficient, and low-performance groups have been widely used to describe the subject group who could not achieve BCI performance at satisfactory levels. To deal with the problem of BCI illiteracy appropriately, previous studies have attempted to design more efficient BCI paradigms (Jeunet et al., 2016; Abiri et al., 2019) or to discriminate low-performance groups and others prior to the main BCI tasks (Bamdadian et al., 2014; Suk et al., 2014). Many past studies have focused on finding capable predictors for the following BCI performance. If the individual degree of the subsequent BCI performance can be estimated, much time and resources can be saved (Blankertz et al., 2010; Ahn et al., 2013; Bamdadian et al., 2014; Suk et al., 2014; Kwon et al., 2020).

The idle EEG signals recorded during prior resting states in the eyes-open (REO) or eyes-closed (REC) condition can be used to predict subsequent BCI performance (Hammer et al., 2012). For example, Blankertz et al. (2010) have proposed a specific predictor of BCI performance as a set of hyperparameters of the power spectral density (PSD) functions covered with sensorimotor rhythms recorded at two central regions during the REO condition. Another study used the normalized theta band power during the REO condition as a BCI-performance predictor by performing a binary classification with a filter-bank common spatial pattern and support vector classifier (Bamdadian et al., 2014). Ahn et al. (2013) demonstrated that BCI performance was highly related to the proposed performance potential factor (PPfactor) as a combination of high theta (4–8 Hz) and low alpha (8–13 Hz) powers during the REO condition. A recent follow-up study has modified the PPfactor proposed in Ahn’s study by considering both REO and REC conditions (Kwon et al., 2020). Apart from the well-known spectral characteristics of sensorimotor rhythms recorded from a specific motor-relevant area, there is also a line of research that aims to reveal the neural mechanisms underlying motor imagery (MI) processes through large-scale brain connectivity analysis between multiple EEG signals and to find some useful predictors of subsequent BCI performance from the neural dynamics of the brain network (Zhang et al., 2015; Corsi et al., 2020). Because there is no actual movement, but only imagination, the cognitive process for MI is known to be highly related to motor-related neural substrates, and comparative studies have attempted to reveal the common (Holmes and Collins, 2001) or different motor-related cognitive networks (Glover and Baran, 2017) compared to motor execution or preparation (Hardwick et al., 2018; Glover et al., 2020; Van der Lubbe et al., 2021). For example, a study has performed dynamic causal modeling (DCM) to delineate dynamic changes in the effective connectivity of EEG sensorimotor rhythms, including the mu and beta bands, across four regions of interest, such as the primary motor cortex (M1), supplementary motor area (SMA), premotor cortex (PMC), and dorsolateral prefrontal cortex (DLPFC). By investigating significant changes in the connectivity strength between the DLPFC and others, it was shown that the DLPFC is a part of a critical area, not only in various cognitive processes, but also in motor-related cognitive processes (Kim et al., 2018). In addition, recent studies have addressed the significant relationship between the characteristics of brain connectivity and subsequent BCI performance (Phang and Ko, 2020; Vidaurre et al., 2020). For example, Vidaurre et al. (2020) have studied some specific characteristics of functional connectivity between the motor and somatosensory systems by performing imagery coherence analysis of the EEG sensorimotor rhythm, including the mu and feedback bands recorded during the MI task with an online feedback paradigm. By investigating ipsilateral (within) and contralateral (between) functional connectivity between the primary motor and somatosensory cortices, the authors showed that the within connectivity strength was positively correlated (*r* = 0.31) with BCI feedback accuracy and proposed that this connection could be a predictor of subsequent BCI performance (Vidaurre et al., 2020). Another study has calculated the functional connectivity of sensorimotor rhythm as alpha and beta bands recorded during a binary classification MI task. Based on the parietal area centered on the contralateral parietal regions (P3/5/6), investigation of the characteristics of EEG alpha (8–13 Hz) rhythms within the parietal network and between others has shown that the subsequent BCI performance is positively correlated with the connectivity strength of the intralobular parietal network (*r* = 0.31), but were negatively correlated with the interlobular parietal network (*r* = −0.34) (Phang and Ko, 2020). Similar to studies on the spectral characteristics of single EEG signals with the subsequent BCI performance, there is a series of EEG studies on the characteristics of dynamic brain connectivity in the resting state (Zhang et al., 2015; Lee et al., 2020; Yoon and Lee, 2020). For example, Zhang et al. (2015) investigated the relationship between MI classification accuracy and several network measurements of the brain network constructed by spectral coherence among multivariate EEG signals within 4–14 Hz recorded during the resting state with eyes closed condition. By showing the positively and negatively correlated network measurements with the MI classification accuracy, the authors proposed that the intrinsic characteristics of functional connectivity of the EEG signals in resting states could be used as predictors for the subsequent BCI performance (Zhang et al., 2015).

As mentioned above, various spectral and brain connectivity studies have been conducted on resting state EEG signals for accurate prediction and significant relation with individual ability to upcoming BCI performance. However, most of these methodologies was based on a linear model in which the EEG signal to be analyzed is stationary. It is worthy adopting a non-linear analysis to the subsequent BCI performance because the EEG signal is a non-stationary signal and is successfully used in clinical and cognitive psychology research that mainly deals with individual or group differences in specific difference conditions. For this reason, this study mainly investigated the feasibility of using non-linear EEG features for the problem of BCI illiteracy. Specifically, we attempted to adopt several well-known non-linear EEG features such as sample entropy (Richman and Moorman, 2000), permutation entropy (Bandt and Pompe, 2002), and recurrence quantification analysis (RQA) (Marwan et al., 2007) to achieve this. In various EEG research fields, these non-linear approaches have been successfully used to characterize conscious and sleep stages (Kreuzer et al., 2014; Rolink et al., 2015; Shabani et al., 2016; Mateos et al., 2018) and to find a significant relationship between several brain disorders such as epilepsy (Niknazar et al., 2013), autism (Heunis et al., 2018; Kang et al., 2019), and schizophrenia (Takahashi et al., 2010; Hauge et al., 2011). Non-linear EEG analysis, thus, could provide very useful features for tracing the changes or discriminating the individual differences in the intrinsic properties of EEG signals between specific groups or individuals. In contrast to the common use of non-linear EEG features in clinical and neuroscience research, the availability of these features in BCI application systems has remained relatively insignificant. Because, more computing time and resources are required to extract non-linear features than simple spectral-based features, it is not suitable for online BCI application systems that require fast response. However, recent developments in computer hardware and efficient algorithm technology, have led to a gradual increase in the use of non-linear features in offline BCI research. In particular, some studies that actively utilized the RQA measurements for BCI- or motor-related systems are reported below. Recent EEG-BCI research has measured the similarity between pairwise EEG electrodes by using one of the RQA measurements, space-time recurrence (STR). By calculating the STR between all EEG pairs, the authors constructed a specific adjacency matrix for estimating functional connectivity over all EEG sites. They showed the availability of these RQA approaches to distinguish MI tasks in the BCI context (Rodrigues et al., 2019). Another EEG study conducted the RQA to estimate the specific temporal changes in the regularity and complexity during motor execution tasks. By showing the temporal changes in two RQA measurements as determinism (DET) and the recurrence time entropy (RTE), the authors addressed that the complexity of EEG signals significantly modulated by the execution of real motor tasks (Pitsik et al., 2020). In a more relevant study on BCI applications, by using a steady-state motion visual evoked potentials (SSMVEP)-based BCI paradigm, Gao and colleagues addressed the significant differences in two RQA network measurements, the weighted local efficiency and clustering coefficients, from the multivariate weighted recurrence network across five EEG literates and five EEG illiterates (Gao et al., 2019). The studies introduced above show good examples of RQA usage for the BCI systems. However, to the best of our knowledge, no study to date has addressed the use of non-linear measurements as predictors of the subsequent MI-BCI performance or BCI applications. Another important point is that most of the significant evidence revealed by past studies was obtained from relatively simple linear classifiers, such as linear discriminant analysis (LDA) or a support vector machine (SVM) based on online BCI tasks. From the perspective of the implementation of real BCI applications focused on low cost, it is logical to adapt these simple and light approaches. Past studies on individual EEG characteristics of BCI performance did not use deep learning technology because of its high computational cost. However, many studies have recently adopted the convolutional neural network (CNN)-based (Schirrmeister et al., 2017; Lawhern et al., 2018; Zhang et al., 2018) or long short-term memory (LSTM)-based (Wang et al., 2018) classifiers to decode the specific intentions in the MI-BCI task (Craik et al., 2019; Roy et al., 2019). From the perspective of the original goal of dealing with the low-performance BCI groups intensively, decoding approaches using deep learning should be considered in order to reveal the relationship between BCI performance and some individual EEG characteristics or predictors in the prior states. In particular, it is necessary that the innate and invariant EEG ability of individuals be extracted by various types of decoding methods in order to determine their influence on BCI performance.

To overcome the limitations of linear spectral-based approaches and simple classifiers, this study aimed to solve the problem of identifying a low-performance BCI group by adopting non-linear EEG features in the prior state and deep learning decoding methods in the subsequent BCI tasks. Two types of EEG signals – recorded at the prior resting state and for MI tasks – were separately processed. For the prior resting states, non-linear analyses were mainly used to generate EEG features for the intrinsic properties of an individual. For subsequent BCI tasks, decoding methods based on traditional and CNN-based classifiers were mainly used to quantify the EEG ability of performing relevant MI tasks. The results demonstrate that RQA can estimate upcoming BCI performance by indicating the significant relationship between the RQA measurements derived from the prior resting state and the subsequent BCI performance.

## Materials and Methods

### EEG Motor Movement/Imagery Database

This study used the EEG motor movement/imagery database (EEGMMIDB) (Schalk et al., 2004) which is publicly accessible via Physionet (Goldberger et al., 2000) because it is suitable for the goal of this study, which is to investigate the relationship between two different kinds of EEG signals recorded in the prior resting state and the subsequent BCI tasks. Most importantly, a relatively large number of subjects (109) were recruited from this database compared to other BCI databases. In the EEGMMIDB, all EEG signals were commonly recorded in accordance with 14 individual experimental blocks, where the first two blocks correspond to one-minute REO and REC conditions, respectively, and the remaining blocks were four different types of binary tasks: (1) open and close left or right fist, (2) imagine opening and closing left or right fist, (3) open and close both fists or both feet, and (4) imagine opening and closing both fists or both feet, three times. This study solely focused on the signals during motor imagery tasks so that we used only two imagery tasks as (2), (4) and ignored the real execution tasks as (1), (3). All MI trials were identically conducted during a fixed period from 0 to 4 s with the prior 2-s fixation duration based on the onset of MI action cue. All EEG signals were recorded with the BCI2000 system using 64 channels sampled at 160 Hz according to the international 10/10 layout system. We removed the data recorded from five subjects in which there was some serious fluctuation or data missing in the signals to prevent reliable EEG analyses. In addition, we used only 56 channels out of the 64 EEG channels, eliminating the redundant eight channels (AF7/8, FT7/8, T9/10, Iz, Oz) that were also removed in the relevant past studies due to superfluous motion artifacts (Rocca et al., 2014; Kang et al., 2018). To investigate the relationship of the EEG features from the prior resting state with the BCI performance in the following BCI tasks, individual EEG signals in the EEGMMIDB were divided into resting states and binary MI-BCI tasks. And then, these two distinct parts of the EEG signal were separately processed with individual pipelines for the relevant EEG analyses after simple preprocessing. Broadly, the EEG signals in the prior resting state were converted into three distinct groups of EEG features to represent the intrinsic EEG properties of individual subjects. By contrast, the EEG signals in the MI-BCI tasks were decoded to the specific intention of the corresponding MI tasks by four different decoding methods. For the assessment of EEG capability of individual subjects, we finally quantified the subsequent BCI performance by maximizing the results of four different methods.

### Resting State

The prior resting states in the EEGMMIDB consisted of two one-minute EEG signals during the REO and REC conditions. To characterize these parts of EEG signals efficiently, we converted the signals into a group of EEG features by performing a linear spectral analysis and three types of non-linear analyses in both REO and REC conditions separately. Each analysis generated a corresponding set of EEG features that represented the intrinsic properties of individual EEG signals. Prior to these EEG analyses, both one-minute EEG signals were band-pass filtered with frequencies ranging from 0.1 to 55 Hz. These procedures for EEG filtering are commonly conducted using linear-phase finite impulse response (FIR) filters with forward and backward operations to avoid phase distortion and edge effects. Next, the filtered EEG signals were randomly segmented into 30 2-s epochs. Note that all following EEG procedures for both REO and REC conditions in this study were performed in an epoch. Subsequently, all EEG features extracted from all epochs were pooled and finally constructed into a set of EEG features by calculating the average or standard deviation across all epochs.

#### Spectral Power

In the spectral analysis, a fast Fourier transform (FFT) with a Hamming window estimated the power spectral density (PSD) of all resting state EEG signals in an epoch. The median frequency and Shannon entropy of the PSD were also calculated. The frequency range of PSD was bounded to 4 and 50 Hz and then separated into the four distinct band powers (BPs) by averaging across the corresponding frequency ranges [theta (θ): 4–8 Hz; alpha (α): 8–13 Hz; low beta (*L*β): 13–20 Hz; high beta (*H*β): 20–30 Hz]. And then, 10 ratios of BPs from all possible pairs were calculated from these BPs. To sum up, a procedure of linear spectral analysis converted the prior resting state EEG signals into 12 EEG features such as a median frequency, a Shannon entropy, and 10 ratios of BPs.

#### Sample Entropy

As one of the well-known linear analysis for EEG signals, sample entropy (SE), which was proposed by Richman and Moorman (2000), has been widely used to represent the complexity of time series. Similar to another entropy-based non-linear complexity measurement called approximate entropy (Pincus et al., 1991), the sample entropy indicates the regularity and similarity of a time series. However, sample entropy has two significant advantages over approximate entropy: bias reduction and consistency, independent of data length (Richman and Moorman, 2000). Sample entropy is defined as the negative logarithm of the conditional probability that two sequences that are similar to each other up to *m* points remain similar within a tolerance *r* at the (*m+1)*-th point, except for the self-match condition. Given *N* data points, a template vector is defined that starts at a specific point *i* and has a length *m* such that *x*_*m*_(*i*) = {*x*_*i*_,*x*_*i* + 1_,*x*_*i* + 2_,⋯,*x*_*i* + *m*−1_}. Next, the distance between two vectors, *d*[*X*_*m*_(*i*),*X*_*m*_(*j*)], is obtained by calculating the maximum absolute difference of their corresponding scalar components and counting the number of template vector pairs that are matched within *r*. Then, the sample entropy can be expressed as

(1) $$S\,E\,\left(m\right)=-\log\left(\frac{P_{m+1}}{P_{m}}\right),$$

where *P*_*m+1*_ is the probability that two sequences will match for the (*m+1*)-th point, and *P_m_* is the probability that two sequences match for *m* points. In this study, *N* was 320 as the number of points in a 2-s EEG epoch. In the parameters for SE calculation, we set *m* as 1, and *r* was 0.2.

#### Permutation Entropy

Permutation entropy (PE) proposed by Bandt and Pompe (2002) quantifies the complexity of a time series based on the number of times that similar symbolic motifs occur in the phase space. Compared to other non-linear quantities, PE can be calculated more easily owing to the model-free method with the concept of symbolic sequences (Staniek and Lehnertz, 2008). Given an embedded signal *X_j_* with the embedding dimension (*D*) and time delay (τ), the elements of *X_j_* are arranged in the ascending order {*x*_*j* + (*i*_1_−1)τ_ ≤ *x*_*j* + (*i*_2_−1)τ_ ≤ ⋯*x*_*j* + (*i*_*D*_−1)τ_}, where *i*_*D*_ denotes the index of the largest element in *X*_*j*_, *i*_(_*_*D*_*_–1)_ is the second largest, and so forth. Then, each vector *X_j_* with the resulting index vector [*i*_1_,*i*_2_,⋯,*i*_*D*_] is mapped to a single motif among *D*! possible permutations. Let *f*(π_*i*_) denote the frequency of the *i*-th motif (denoted by π_*i*_) in a time series. Then, the probability distribution of distinct motifs is defined as:

(2) $$p\left(\pi_{i}\right)=\frac{f\,\left(\pi_{i}\right)}{\sum_{i=1}^{D!}f\,\left(\pi_{i}\right)}\;fori=1,..,D!.$$

Having the probabilities of all of the motifs, the PE is defined as

(3) $$H=-\sum_{i=1}^{D!}p\,\left(\pi_{i}\right)\,\ln p\,\left(\pi_{i}\right).$$

Finally, the PE is normalized in the range between 0 and 1:

(4) $$0\leq P\,E=\frac{H}{\ln\left(D!\right)\leq 1},$$

where PE = 0 when there is only one motif in the time series, and PE = 1 when all of the motifs occur with equal probabilities. For the parameters of PE calculation in this study, we used the *D* as 3 and τ as 1.

#### Recurrence Quantification Analysis

To derive some useful non-linear dynamic properties from the prior resting state EEG signal, we also performed a recurrent quantification analysis (RQA). Contrary to the two entropy-based non-linear analyses described above, RQA is fundamentally based on the concept of recurrence in a dynamic system (Marwan et al., 2007). Recurrence refers to the trajectory returning to the former state in the phase space, which is generally constructed from a time-series signal using a time-embedding method. A recurrent plot (RP), which was proposed by Eckmann et al. (1987), was used to visualize the amount of recurrence in a multidimensional dynamic system by simply illustrating a white-black dot square matrix into a two-dimensional space. According to Eq. 5., RP is calculated for each sample *i*, *j* of time series *x*, under the predefined threshold distance ε.

(5) $$R_{i,j}=\Theta \,\left(\varepsilon -||x_{i}-x_{j}||\right),i,j=1,2,\ldots \,N,$$

where Θ(⋅) is the Heaviside function, |⋅| is the maximum norm, and *N* is the number of samples in the phase-space trajectory. That is, if the distance in the phase space between *x_i_* and *x_j_* falls within the ε, two samples are considered to be recurrences, indicated as *R*_*i,j*_ = 1 (black); otherwise, *R*_*i,j*_ = 0 (white) in an *N*×*N* RP matrix. Based on the constructed black-white dot square matrix of RP, RQA was developed to characterize various types of graphical patterns in RPs by defining a group of RQA measurements. For example, RQA quantified not only the density of recurrence points but also the histograms of the lengths of the diagonal and vertical lines in an RP. For the procedures of RQA, we fixed the ε as 0.3 according to previous RQA studies (Ngamga et al., 2016; Yang et al., 2018). By using pyRQA toolbox (Rawald et al., 2017), we calculated three RQA measurements as determinism (DET), Laminarity (LAM), and Mean recurrence time (MRT). Compare to other RQA measurements, these representative measurements were widely used in neural signal analysis because it could be intuitively explained as the characteristic of signal properties. First, DET is defined as the fraction of recurrence points which from a diagonal line of minimal length (I_*min*_), as follows:

(6) $$\sum_{l=l_{m\,i\,n}}^{N_{l}}l\,P\,\left(l\right)/\sum_{l,j=1}^{N_{l}}R_{i,j},$$

where *P*(*l*) denotes the frequency distribution of the length *l* of the diagonal line in the RP. As it quantifies the predictability of the system, the DET value tends to 0 for the chaotic system, while it is equal to 1 for the periodic system. Second, LAM is defined as the histogram of the lengths of the vertical lines as follows:

(7) $$\sum_{v=v_{m\,i\,n}}^{N_{v}}v\,P\,\left(v\right)/\sum_{l,j=1}^{N_{l}}R_{i,j}.$$

Because it calculates the relative number of vertical patterns over the entire RP, in turn, it represents slowly changing states and, thus, the occurrence of laminar states in the system. LAM will decrease if the RP contains recurrent points that are more isolated than in vertical or diagonal structures. The mean recurrence time (MRT) is the average length of white vertical lines in the RP as follows:

(8) $$\sum_{w=1}^{N_{w}}w\,P\,\left(w\right)/\sum_{w=1}^{N_{w}}P\,\left(w\right),$$

where *p*(*w*) denotes the frequency distribution of the lengths w of white vertical line. It mainly defines the temporal variations of the time-series signals by measuring for harmonic oscillations in the corresponding the period length (Gao, 1999). In this study, we set the I_*min*,_ V_*min*_ as 2.

### BCI Performance

Contrary to the procedures for EEG feature extraction in the resting state, the EEG signals recorded in the MI tasks were decoded to determine the type of motor intention in the signals. To do this, four different decoding methods were used in this study. All methods are proposed to classify two goal-directed intentions based on the corresponding binary MI tasks in the EEGMMIDB. The EEG signals of MI tasks had a fixed period from −2 to 4 s that consisted of 2-s fixation and 4-s MI task durations. Prior to the EEG decoding analyses, the EEG signals during the MI tasks were first segmented into 6-s epochs, and 4-s signals in the BCI task had zero mean values by subtracting from the mean of 2-s fixation. All the following decoding methods were conducted using zero-mean EEG signals corresponding to the 4-s MI duration. For the binary classification test, all the decoding methods, which will be described in sequence, were conducted using a 5 × 5-fold cross-validation test. In this test, all the EEG trials during the MI tasks were randomly segmented into training and test sets; training and testing were done for four and one epochs, respectively. This process was repeated five times by randomizing the order of trials.

#### Classical Decoding Approach – FBCSP

First, we adopted the filter bank common spatial pattern (FBCSP) method proposed by Ang et al. (2012). This method is based on the CSP analysis, which is intended to find the optimal spatial filter that maximizes the variance of signals in one condition and minimizes the variance of signals in another condition at the same time. In FBCSP, the EEG signals containing specific goal-directed motor intentions were filtered and decomposed into a set of specific band-pass signals by the corresponding linear-phase FIR filters. This study also adopted a total of nine band-pass filter banks: 4–8, 8–12,…, 36–40 Hz, like the previous study (Ang et al., 2012). Specifically, assuming *W_b_* as a projection matrix of the spatial filter in the specific *b*th band-pass filtered signal at the *i*th trial after spatial filtering is defined as

(9) $$Z_{b,i}=W_{b}^{T}\,E_{b,i},$$

where *Z*_*b,i*_, *E*_*b,i*_ are the CSP spatial filtered signal and the band-pass filtered EEG signal in the *i*th trial and the *b*th band, respectively. The projection matrix can be obtained using the following equation:

(10) $$\sum_{b,1}W_{b}=\left(\sum_{b,1}+\sum_{b,2}\right)\,W_{b}\,D_{b,1},$$

where _*b,1*_ and _*b,2*_ are the covariance matrices of the *b*th band-pass filtered signals under the two conditions, and *D_b_* is the diagonal matrix that contains the eigenvalues of _*b,1*_. That is, the projection matrices of all bands could be calculated in the training session and used to convert the EEG signals to spatially filtered signals that highlight the degree of binary discrimination between the two classes. The CSP features as input matrices for the classifier were obtained using the following equations.

(11) $$V_{b,i}=\log\left(d\,i\,a\,g\,\left({\bar{W}}_{b}^{T}\,E_{b,i}\,E_{b,i}^{T}\,{\bar{W}}_{b}\right)/t\,r\,\left[{\bar{W}}_{b}^{T}\,E_{b,i}\,E_{b,i}^{T}\,{\bar{W}}_{b}\right]\right),$$

where *V*_*b*,*i*_ ∈ *ℝ*^2*m*^; ${\bar{W}}_{b}$ represents the first *m* and the last *m* columns of *W_b_*, *diag*(⋅) is the diagonal element of the square matrix, and *tr*(⋅) is the sum of the diagonal elements in the square matrix. To calculate the CSP feature, *m* was set as 2. Next, the extracted FBCSP features were classified using a quadratic discriminant analysis (QDA) classifier instead of the Naive Bayesian Parzen Window (NBPW) classifier proposed in the original paper (Ang et al., 2012), because the QDA classifier exhibited better performance than the NBPW classifier for the EEGMMIDB.

#### CNN-Based Decoding Approach – ShallowConvNet, DeepConvNet, and EEGNet

We also performed deep-learning approaches to decode the EEG signals using three types of convolutional neural network (CNN)-based classifiers: ShallowConvNet, DeepConvNet, and EEGNet. The first two network models were proposed by Schirrmeister et al. (2017), and the last model was proposed by Lawhern et al. (2018). From the perspective of EEG signal processing, it is likely that these CNN-based methods commonly consist of three types of neural networks that correspond to different roles of signal processing: temporal filtering, spatial filtering, and binary classifiers, although there are different types of layouts in neural networks among these methods. Detailed descriptions and specific parameters in the corresponding layout in the three neural networks are tabulated in the Supplementary Information (Supplementary Tables 1–3). Each CNN method processed the raw EEG signals during the BCI task, and then converted them into specific input features to a binary classifier that implemented by a fully connected network at the end of the corresponding CNN model. Consequently, three individual CNN models conducted the binary classification in a subject, and the results of classification were quantified as binary accuracy in each subject. The dropout rate was set to 0.2, and the activation functions were the exponential linear unit (ELU) in EEGNet and DeepConvNet, rectified linear unit (RELU) in ShallowConvNet, and softmax function in all fully connected networks. The loss functions are the cross-entropy function, and the optimizer is adaptive moment estimation (ADAM). All CNN-based decoding methods were realized by Keras and TensorFlow 2.0.

#### BCI-Performance Groups

Based on all binary accuracies in subjects from the four different decoding methods addressed above, we first defined the subsequent BCI performance which is responsible for the criteria to be investigated with the EEG features obtained from the prior resting state. Because we focused on measuring the degree of implicit EEG information in a subject as maximally as possible, all individual BCI performances were assigned as the maximum accuracy among all results of these four decoding methods. Based on the obtained BCI performance, therefore, we intentionally divided all subjects in the EEGMMIDB into three groups: low-, intermediate-, and high-performance groups according to the following statistical procedures. First, we followed Muller-Putz’s statistical method (Müller-Putz et al., 2008) which enabled us to calculate the significant confidence interval under certain predefined conditions in a balanced dataset, such as the total number of subjects, the number of subjects in a class, and the significance level (*p*-value). Specifically, the unbiased estimator of the mean and confidence intervals is then given as follows:

(12) $$\bar{X}=\frac{1}{N}\,\sum_{i=1}^{N}X_{i}$$

(13) $$\tilde{p}=\frac{N\,\bar{X}+2}{N+4},$$

(14) $$C.I=\tilde{p}\pm \sqrt{\frac{\tilde{p}\,\left(1-\tilde{p}\right)}{N+4}\cdot }\,Z_{1-\frac{\alpha }{2}},$$

where *X_i_* is the individual maximal accuracy rate in the *i*th subject, *N* is the total number of subjects, and *Z* is the standard normal distribution with a significance level α. Typically, α is 0.1, 0.05, or 0.01. In this study, because the number of subjects was 104, the upper values of the two confidence levels at α 0.05 or α 0.01 were obtained as 0.594 and 0.658, respectively. Second, based on these criteria, we divided all subjects into three types according to BCI performance. The high-performance group was above 0.658, the intermediate-performance group was between 0.594 and 0.658, and the low-performance group was below 0.594. Finally, after the discrimination of BCI performance groups, we used the value of subsequent BCI performance as the kappa (*ϰ*) values, which were converted from the maximum accuracy rates according to Eq. 12, because the kappa values were normalized from 0 (chance level) to 1 (perfect classification). For the linear correlation or regression with individual EEG features in the prior states, the converted kappa values would limit the output values by preventing unnecessary bias.

(15) $$\varkappa =\frac{p_{a}-p_{c}}{1-p_{c}}$$

### Statistical Analysis

The individual EEG features in both the REO and REC conditions were analyzed by linear correlation along with analysis of variance (ANOVA) to clarify which EEG features in the resting state strongly affected the subsequent BCI performance. First, one-way ANOVA tests were carried out to investigate the significant differences in individual EEG features across three BCI groups. Due to the unequal size of the BCI groups, the Kruskal-Wallis test was first conducted, followed by the Welch’s *t*-test, and the results were *post hoc* analyzed to individually investigate whether there were significant differences between the high-performance group and the others or the low-performance group and the others. For cases where the statistical results of ANOVA indicated a significant difference (*p* < 0.01), additional linear correlation analyses were conducted to verify the significant linear tendency between the value of individual EEG features in the prior resting state and the subsequent BCI performance based on the absolute values of linear correlations (|*r*| > 0.3). All statistical results exhibited significant differences, satisfying the significance level of 0.01, with Bonferroni-adjusted *p*-values. All EEG features in the prior resting state could be constructed as the average or standard deviation of individual features across all epochs. However, we could not find any meaningful statistical results for a group of EEG features defined as the standard deviations across all epochs. Therefore, all the statistical results were obtained only by means of the average across all epochs in both REO and REC conditions.

## Results

### Decoding Performance in Subsequent BCI Tasks

**FIGURE 1:**
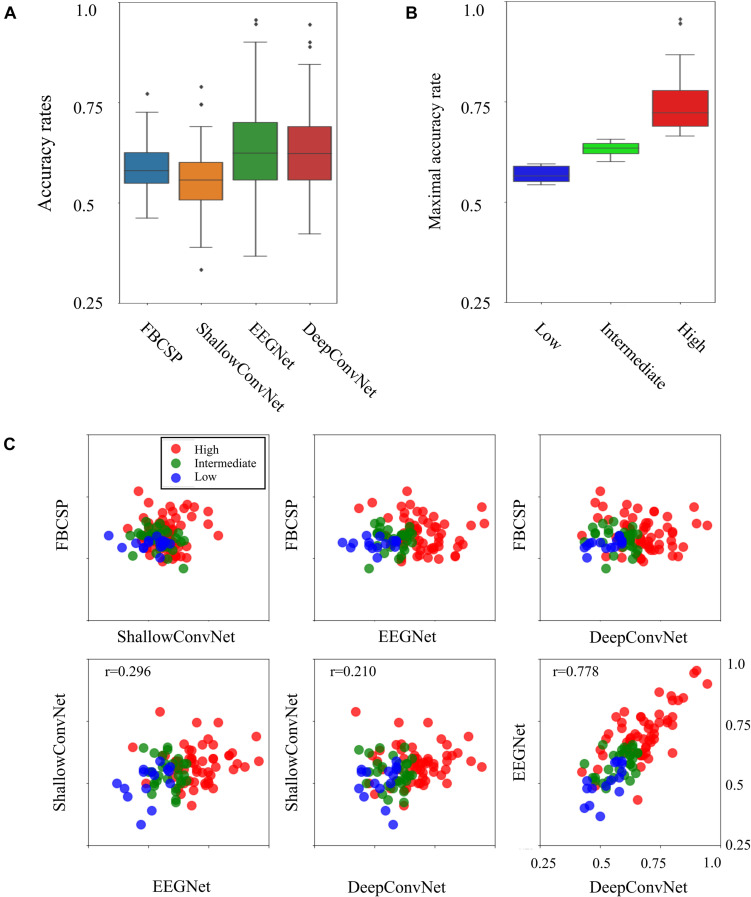
Four decoding results and three BCI groups. **(A)** Comparison of accuracy rates between four different decoding methods. **(B)** Boxplots indicating BCI performance as the maximum accuracy rates across four decoding methods in the three BCI groups. **(C)** Scatterplots of binary classification accuracy rates for all possible pairwise comparisons between two decoding methods. The tick labels of the *x*- and *y*-axes in all the plots are the same as tick labels presented in the right bottom axis.

Four individual decoding methods, FBCSP, ShallowConvNet, DeepConvNet, and EEGNet, were used to calculate the corresponding individual binary accuracy rates for the motor imagery tasks in the subject. Figure 1 compares all individual binary accuracy rates and the differences in maximum accuracy rates, which were the maximum values among four individual classifiers in the subject, between the three types of BCI-performance groups. As shown in Figure 1A, the mean accuracy rates for all the individual decoding methods did not significantly differ from each other in the ANOVA analysis (*p* < 0.01). The means and standard deviations with maximum and minimum accuracy rates for each method were 0.588 ± 0.058 (max: 0.772, min: 0.461) in the FBCSP, 0.556 ± 0.073 (max: 0.789, min: 0.333) in the ShallowConvNet, 0.635 ± 0.116 (max: 0.956, min: 0.367) in the EEGNet, and 0.627 ± 0.109 (max: 0.944, min: 0.422) in DeepConvNet. The six scatter plots in Figure 1C show a comparison of the decoding methods. The Pearson coefficients with statistical significance (*p* < 0.05) were 0.296 between ShallowConvNet and EEGNet, 0.210 between ShallowConvNet and DeepConvNet, and 0.778 between EEGNet and DeepConvNet. All the other comparisons were not statistically significant. Therefore, these results showed that the performances of EEGNet and DeepConvNet were relatively similar to those of the other methods. Figure 1B shows a comparison of the BCI performance between the low-, intermediate-, and high-performance BCI groups. The groups were intentionally divided based on BCI performance, which is indicated by the maximum accuracy rate among all decoding methods. The means and standard deviations of the accuracy rates with the number of subjects in each group were 0.570 ± 0.020, 0.631 ± 0.017, and 0.743 ± 0.073 in the low- (15 subjects), intermediate- (30 subjects), and high-performance (57 subjects) groups, respectively.

### Relationship Between the EEG Features and the BCI Performance

**FIGURE 2:**
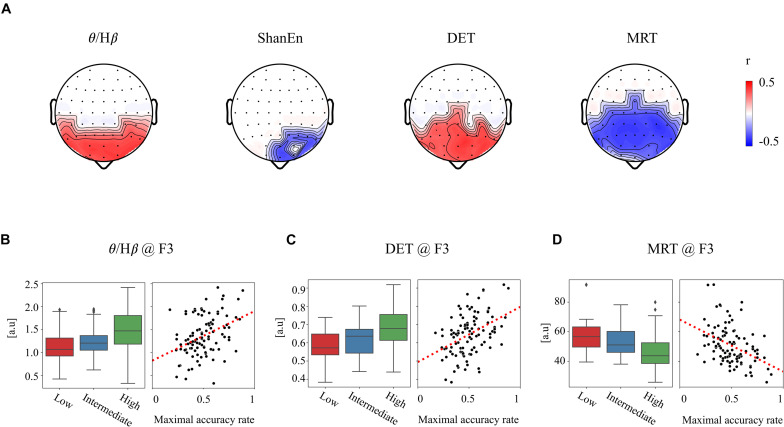
Significant relationship between the prior EEG features and subsequent BCI performance. **(A)** Four exemplar topographies for significant EEG features in the REO condition with significant statistical results (*p* < 0.01 and |*r*| > 0.3). **(B–D)** Three exemplar EEG features at the F3 site corresponding to **(B)** θ/*H*β, **(C)** DET, and **(D)** MRT. Boxplots of each feature corresponding to three BCI groups (left) and scatterplots of each feature corresponding to the BCI performance (right).

First, none of the EEG features in the REC condition showed a significant relationship with subsequent BCI performance. In other words, the EEG features obtained from the REC condition did not have any information related to the subsequent BCI tasks. In contrast, several EEG features derived from the REO condition were highly associated with the subsequent BCI performance. All the significant results of individual EEG features in the REO condition are summarized in Table 1 (no significant results were omitted). Figure 2A illustrates topographically the four representative EEG features in the prior REO resting state, which were highly associated with the subsequent BCI performance. Most spectral EEG features were insignificant. However, compared to other bands or other areas, the ratio of theta to beta power at the frontal area were significantly correlated with the subsequent BCI performance. Another spectral feature, the Shannon entropy in the PSD at the left frontal areas, also had a significant negative correlation with the BCI performance shown in Figure 2A. Considering the non-linear entropy features, both sample entropy and permutation entropy did not show any significant results across all brain areas. In other words, both non-linear entropy-based features were not suitable for the prediction of upcoming BCI tasks. RQA features such as DET, LAM, and MRT were significantly correlated with the subsequent BCI performance. Similar to the ratio of theta to beta power, the topographic distribution of significance in the three RQA features shown in Figure 2A indicated that significant differences were mainly observed on the frontal and central areas.

**TABLE 1:** Significant results of the linear relationship between the prior EEG features in the REO condition and the subsequent BCI performance (p < 0.01, Bonferroni-adjusted p -values; L, H in table are the abbreviations of low-performance and high-performance group, respectively).

Variable	Channel	Linear regression		Others vs. L		H vs. Others		Variable	Channel	Linear regression		Others vs. L		H vs. Others	
									
		Slope	*r*-value	t	dof	t	dof			Slope	*r*-value	t	dof	t	dof
θ/L	FC5	0.14	0.31	2.0	21.9	4.0	98.9	DET	FCz	0.72	0.38	2.9	25.6	3.4	96.9
	FC3	0.13	0.31	1.6	19.6	3.7	97.7		Fp1	0.45	0.41	2.2	21.2	3.9	96.8
	Fp1	0.09	0.41	2.1	28.0	4.6	92.9		Fpz	0.42	0.37	1.9	21.2	3.8	96.3
	Fpz	0.08	0.37	1.4	22.4	4.5	96.3		AFz	0.62	0.44	3.1	27.3	4.4	98.1
	Fp2	0.08	0.36	1.8	22.5	4.0	96.7		AF4	0.40	0.37	2.1	19.9	3.4	94.1
	AF3	0.11	0.41	1.5	23.6	4.1	94.0		F7	0.52	0.42	2.3	21.7	4.1	94.1
	AFz	0.11	0.40	1.6	23.0	4.8	96.5		F5	0.52	0.42	3.0	22.3	3.7	98.7
	AF4	0.10	0.41	2.1	23.4	4.6	96.4		F3	0.58	0.42	3.1	23.7	3.9	98.8
	F7	0.14	0.42	2.3	28.3	5.2	90.8		F1	0.70	0.44	2.9	26.5	4.4	98.7
	F5	0.12	0.39	2.2	23.1	4.5	92.8		Fz	0.68	0.42	2.8	24.6	4.0	95.2
	F3	0.13	0.36	1.8	22.0	4.4	97.4		F2	0.57	0.38	1.9	20.9	3.6	94.3
	F1	0.13	0.35	1.3	21.3	4.5	98.7		F4	0.50	0.37	2.1	19.7	3.4	93.3
	Fz	0.12	0.32	1.1	20.1	4.3	98.3		F8	0.49	0.41	2.5	20.8	3.7	95.6
	F2	0.12	0.33	1.2	20.7	4.1	99.0	MRT	FC5	0.00	−0.39	−2.0	21.6	−3.6	86.5
	F4	0.11	0.34	1.5	21.0	4.2	98.2		FC3	−0.01	−0.47	−2.7	20.2	−4.3	84.4
	F6	0.11	0.38	2.5	26.8	3.7	91.3		FC1	−0.01	−0.43	−2.7	23.1	−3.9	88.1
	F8	0.12	0.42	3.1	29.0	4.9	93.4		FCz	−0.01	−0.45	−2.8	24.3	−4.2	87.5
θ/H	Fp1	0.09	0.45	2.4	26.7	4.1	97.2		FC2	−0.01	−0.45	−3.1	23.8	−4.1	88.2
	Fpz	0.08	0.40	1.9	22.6	3.8	97.9		FC4	−0.01	−0.42	−3.2	22.2	−3.9	89.3
	AF3	0.11	0.43	2.0	23.6	3.7	97.0		C1	−0.01	−0.38	−2.6	22.5	−3.3	82.7
	AFz	0.13	0.45	2.1	20.8	4.3	99.0		Cz	−0.01	−0.37	−2.4	22.3	−3.3	88.3
	AF4	0.11	0.45	2.6	23.7	4.1	97.1		AFz	−0.01	−0.38	−2.0	21.8	−4.1	80.5
	F7	0.14	0.43	2.9	25.0	4.6	98.6		AF4	0.00	−0.38	−2.5	19.4	−3.7	81.9
	F5	0.14	0.42	2.6	22.8	3.9	97.1		F7	0.00	−0.41	−2.2	21.9	−4.3	78.0
	F3	0.14	0.38	2.1	20.3	3.6	98.6		F5	0.00	−0.42	−2.4	18.6	−3.7	88.3
	F4	0.13	0.37	1.8	19.4	3.5	97.5		F3	−0.01	−0.41	−2.3	19.0	−3.9	91.8
	F8	0.13	0.44	4.0	28.6	4.6	97.8		F1	−0.01	−0.43	−2.1	22.0	−4.4	87.4
LAM	Fp1	0.27	0.40	2.2	21.7	3.7	98.2		Fz	−0.01	−0.44	−2.3	22.5	−4.5	84.7
	Fpz	0.25	0.37	1.9	21.0	3.6	96.8		F2	−0.01	−0.43	−2.3	22.0	−4.4	82.0
	AFz	0.35	0.43	3.0	25.5	4.2	98.1		F4	−0.01	−0.43	−2.7	20.1	−4.2	81.6
	F7	0.32	0.43	2.6	22.4	4.0	97.4		F8	−0.01	−0.47	−3.3	22.7	−4.1	90.8
	F5	0.31	0.41	3.2	26.2	3.5	98.7	shanEn	Fp1	−0.22	−0.40	−2.1	20.1	−3.5	93.0
	F3	0.33	0.41	3.4	27.3	3.7	98.8		AFz	−0.29	−0.41	−3.2	29.0	−3.8	98.5
	F1	0.38	0.43	2.8	26.4	4.1	99.0		F5	−0.27	−0.38	−2.9	23.1	−3.5	98.9
	Fz	0.37	0.42	2.9	24.4	3.8	97.3		F3	−0.27	−0.36	−3.2	27.1	−3.7	98.7
	F8	0.29	0.41	2.7	21.3	3.6	97.2		F1	−0.27	−0.34	−2.4	25.9	−3.7	99.0

**FIGURE 3:**
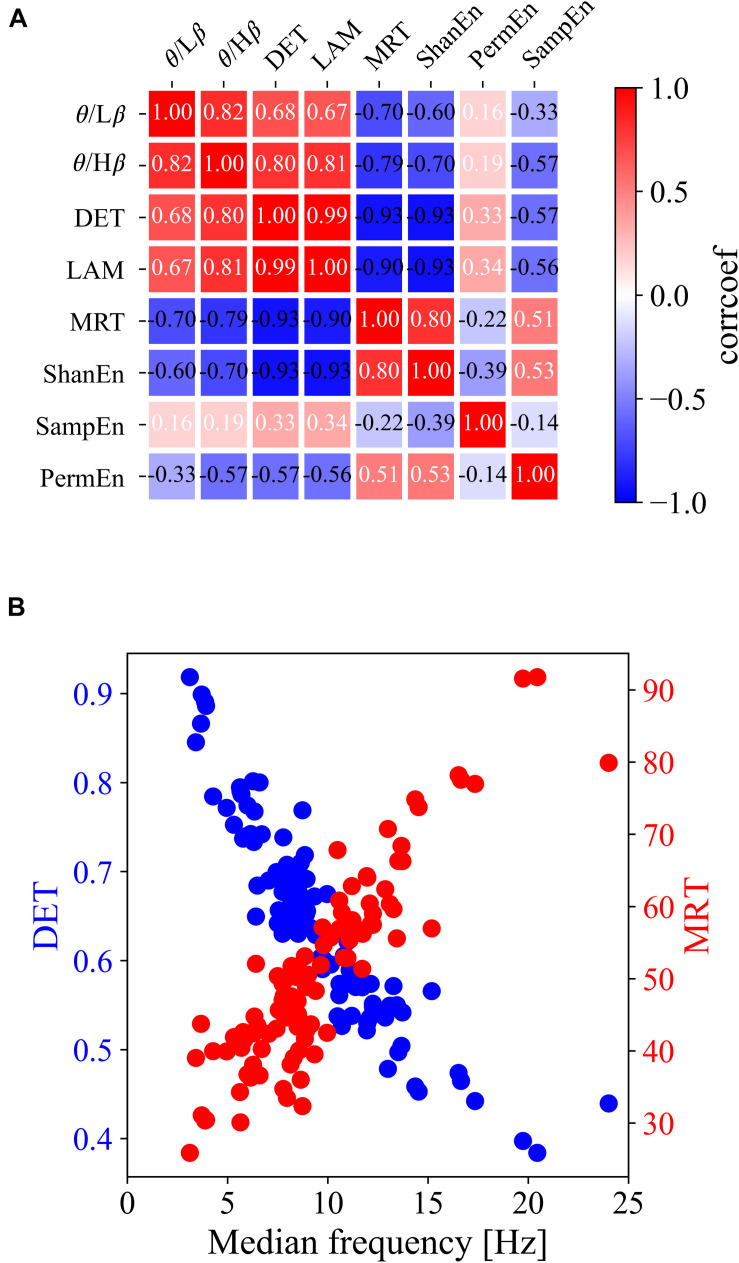
Linear relationship among all types of prior EEG features at the F3 site. **(A)** Matrix of linear correlation coefficients between all pairs of EEG features. **(B)** Scatterplots of the DET and MRT with median frequency.

For clarification, Figures 2B–D show the group differences and linear relationships of subsequent BCI performances with the exemplar EEG features extracted from the left frontal region (F3) in prior resting states. The left-hand side panel illustrates the bar plots with the levels of maximum and minimum values in the corresponding three BCI groups, and the right-hand side panel shows the linear relationship with the subsequent BCI performance. In the ratio of theta to beta power at F3 shown in Figure 2B, the means and standard deviations were 1.497 ± 0.434 in the high-performance group, 1.234 ± 0.346 in the intermediate group, and 1.149 ± 0.460 in the low-performance group. The linear correlation value was 0.38. Figure 2C shows a significant relationship at the F3 site between the DET and BCI performance. Figure 2D shows the corresponding results of MRT at the same F3 site. According to these results, the brain rhythms at the frontal areas were highly related with the subsequent BCI performance. As shown in Table 1, four EEG features, two ratios of band powers and two RQA features (DET, LAM), were positively correlated with the subsequent BCI performance, whereas two EEG features, Shannon entropy and an RQA feature (MRT), were negatively correlated. According to these statistical results, the EEG features were strongly correlated or there was some effect on the frequency of EEG oscillations. To verify these results, linear correlation analyses were performed among the EEG features, and all the correlation coefficients were plotted, as shown in Figure 3A. These results showed that DET and LAM were positively correlated, and MRT was negatively correlated with other RQA features. In Figure 3B, the strong relationship of median frequency between two main RQA measurements is depicted as the negative correlations of the DET and the positive correlation of the MRT. Considering this evidence, we suggest that the predictability of subsequent BCI performance is highly associated with specific EEG characteristics in the prior resting state EEG under the REO condition, in which the enhanced low oscillations (theta rhythm) at the frontal area reduce the non-linear complexity of EEG signals. DET and LAM indicate the degree of complexity in a signal, whereas MRT indicates the degree of predictivity in a signal. That is, if a signal is more chaotic and unpredictable, the DET and LAM values decrease and the MRT value increases, and vice versa.

## Discussion

In this study, we intensively investigated a group of spectral and non-linear EEG features in the resting state. We revealed that both spectral and RQA features extracted from the REO condition before the BCI task were highly correlated with the subsequent BCI performance. Comparing two distinct resting conditions, REO and REC, our results apparently indicated that the EEG features of the REC condition were more highly correlated with the subsequent BCI performance than those of the REO condition. We thought that the dominance of alpha rhythm that drastically occurred during the eyes closed states would contaminate the critical information in the sensorimotor rhythm and other bands. In the spectral analysis, the subsequent BCI performance was closely associated with a lower (theta frequency) rhythm rather than a higher (above beta rhythm) rhythm over the frontal area.

Furthermore, it should be noted that this study mainly addressed the significant relationship between RQA features and the subsequent MI-BCI tasks. It was observed that some groups with higher DET, LAM, and lower MRT in the frontal and central areas were able to achieve higher BCI performance in the subsequent MI-BCI tasks. The linear correlation analysis also showed a significant relationship of the median frequency among these RQA features in which it was positively correlated with both DET and LAM and negatively with MRT. Based on this evidence, we confirmed that the higher performance BCI group had a specific EEG characteristic in which more stable and period dynamic patterns were derived from enhanced theta oscillations in the frontocentral area because both DET and LAM indicate the regularity of a time-series signal; but, the MRT indicates the complexity of a signal.

Despite some spatial differences in topographic distribution, both spectral and RQA results in this study consistently addressed the effect of frontal theta oscillations in the idle resting state on the subsequent BCI performance. More specifically, it should be noted that the enhancement of theta oscillations in the frontal area largely entailed the regular and periodic characteristics of the EEG signals over the frontocentral areas. We believe that these findings apparently indicated the critical role of frontal regions in the idle resting state, whereas the ERD/ERS properties of sensorimotor rhythm during the main BCI tasks directly affected the real BCI performance.

Our main findings indicated a significant relationship between the intrinsic property of frontal theta rhythms in the prior resting state and the subsequent BCI performance. Some subjects who held regular and stable theta activities in the frontal area would be able to perform subsequent MI-BCI tasks. We conjecture the neural mechanism underlying this facilitation of frontal theta activity on the MI-BCI performance as follows: First, it is well known that both ME and MI tasks modulate not only local neural activation in specific motor-relevant regions as somatosensory areas, but also global brain connectivity between other motor-irrelevant regions (Kim et al., 2018; Corsi et al., 2020). More specifically, the frontal regions such as the medial prefrontal cortex (MPFC), dorsolateral prefrontal cortex (DLPFC), and anterior cingulate cortex (ACC) plays very important roles in motor-related cognitive processes with functional connections with other regions (Hardwick et al., 2018; Kim et al., 2018; Van der Lubbe et al., 2021). Interestingly, it has been proposed that the frontal region is closely associated with the default mode network (DMN) in resting states, and the frontal theta rhythm plays a major role in this connection (Scheeringa et al., 2008). In the idle resting state, EEG theta activity in the frontal regions was negatively correlated with BOLD activity in the DMN. An increase in frontal theta activity indicated a decrease in BOLD activity in the DMN, which implied DMN inactivation. In addition, a recent EEG study on effective connectivity between the frontal regions and DMN in the resting state reported that there was significant causal interaction from the MPFC to DMN in the idle resting state and some groups who highly sustained the strong connectivity within the frontal regions, such as the MPFC, DLPFC, SMA, and ACC, facilitated the subsequent BCI performance (Yoon and Lee, 2020). Similarly, according to previous studies on the subsequent BCI performance with EEG functional connectivity, better BCI performance would be observed in conditions where there was higher connectivity within motor-relevant areas, but remained relatively less connected between motor-irrelevant areas (Corsi et al., 2020; Lee et al., 2020; Phang and Ko, 2020). Based on the evidence from previous studies, we proposed that our results in this study showed a strong effect of the frontal region in the resting state on the subsequent BCI performance. Consequently, more inactivated DMN functionally connected with the frontal region via theta oscillations facilitated the subsequent MI-BCI tasks. The corresponding critical clues underlying this neural mechanism would be sufficiently represented as RQA measurements.

This study proposed the critical role of the frontal area in the prior resting state to estimate the subsequent BCI performance; however, there are still several limitations to be revealed in further studies.

First, this study used only the EEGMMIDB to adopt the prior resting state with both REO and REC conditions, and the subsequent MI-BCI tasks in which the EEG signals over the whole brain were obtained from a relatively large number of subjects. However, our results that the availability of RQA as a predictor of MI-BCI performance should be verified again by other types of large-scale EEG databases. Therefore, our next study will follow the various uses of non-linear EEG analysis in BCI applications by including the additional different types of EEG databases to improve BCI illiteracy. Second, despite of our inferences based on the past studies addressed in the discussion, there is still a lack of understanding of the neural mechanisms underlying the regularity and stability of the frontal theta rhythms. Further studies that would use several different types of EEG databases will adopt the source localization and non-linear functional connectivity to reveal the causal relationship of the frontal regions between other motor-irrelevant regions via frontal theta rhythm, not only based on the MI-BCI paradigm but also general EEG ability to BCI application in subjects.

## Conclusion

This study showed that a group of RQA features extracted from the REO condition was highly reflected at the individual level of BCI performance. Both DET and MRT obtained from RQA indicated that more regular and periodic theta rhythms enhanced in the frontal regions during the prior resting state led to higher performance during the subsequent MI-BCI tasks. In conclusion, we address that the evidence in this study suggests the availability of RQA for further BCI applications.

## Data Availability Statement

The original contributions presented in the study are included in the article/Supplementary Material, further inquiries can be directed to the corresponding authors.

## Ethics Statement

Ethical approval was not provided for this study on human participants because the human EEG data in this study was originated from open-accessible database (https://physionet.org/content/eegmmidb/1.0.0/). Written informed consent for participation was not required for this study in accordance with the national legislation and the institutional requirements.

## Author Contributions

J-HK and JK contributed to the conception and design of the study. S-HK performed the statistical analysis. J-HK wrote the first draft of the manuscript. J-HK, S-HK, JY, and JK wrote sections of the manuscript. All authors contributed to manuscript revision, read, and approved the submitted version.

## Conflict of Interest

The authors declare that the research was conducted in the absence of any commercial or financial relationships that could be construed as a potential conflict of interest.

## Publisher’s Note

All claims expressed in this article are solely those of the authors and do not necessarily represent those of their affiliated organizations, or those of the publisher, the editors and the reviewers. Any product that may be evaluated in this article, or claim that may be made by its manufacturer, is not guaranteed or endorsed by the publisher.
