# Supplementary material for: Effects of Frontal Theta Rhythms in a Prior Resting State on the Subsequent Motor Imagery Brain-Computer Interface Performance
Source: Front Neurosci. 2021 Aug 13;15:663101. doi: 10.3389/fnins.2021.663101 (PMC8414888; doi:10.3389/fnins.2021.663101)
Supplement: Supplementary file 1 [file Table_1.docx]

Table S1. ShallowConvNet

| Description | Layers | Shape |
| --- | --- | --- |
| Temporal filter | InputLayer | (None, 56, 480, 1) |
|  | Conv2D | (None, 56, 480, 8) |
|  | BatchNormalization | (None, 56, 480, 8) |
| Spatial filter  (Convolution block 1) | DepthwiseConv2D | (None, 1, 480, 16) |
|  | BatchNormalization | (None, 1, 480, 16) |
|  | Activation ('relu') | (None, 1, 480, 16) |
|  | AveragePooling2D | (None, 1, 120, 16) |
|  | Dropout | (None, 1, 120, 16) |
| Spatial filter  (Convolution block 2) | SeparableConv2D | (None, 1, 120, 16) |
|  | BatchNormalization | (None, 1, 120, 16) |
|  | Activation ('relu') | (None, 1, 120, 16) |
|  | AveragePooling2D | (None, 1, 15, 16) |
|  | Dropout | (None, 1, 15, 16) |
| Binary classifier | Flatten | (None, 240) |
|  | Dense | (None, 2) |
|  | Activation ('softmax') | (None, 2) |

Table S2. DeepConvNet

| Description | Layers | Shape |
| --- | --- | --- |
| Temporal filter | InputLayer | (None, 56, 480, 1) |
|  | Conv2D | (None, 56, 476, 25) |
| Spatial filter  (Convolution block 1) | Conv2D | (None, 1, 476, 25) |
|  | BatchNormalization | (None, 1, 476, 25) |
|  | Activation ('elu') | (None, 1, 476, 25) |
|  | MaxPooling2D | (None, 1, 238, 25) |
|  | Dropout | (None, 1, 238, 25) |
| Spatial filter  (Convolution block 2) | Conv2D | (None, 1, 234, 50) |
|  | BatchNormalization | (None, 1, 234, 50) |
|  | Activation ('elu') | (None, 1, 234, 50) |
|  | MaxPooling2D | (None, 1, 117, 50) |
|  | Dropout | (None, 1, 117, 50) |
| Spatial filter  (Convolution block 3) | Conv2D | (None, 1, 113, 100) |
|  | BatchNormalization | (None, 1, 113, 100) |
|  | Activation ('elu') | (None, 1, 113, 100) |
|  | MaxPooling2D | (None, 1, 56, 100) |
|  | Dropout | (None, 1, 56, 100) |
| Spatial filter  (Convolution block 4) | Conv2D | (None, 1, 52, 200) |
|  | BatchNormalization | (None, 1, 52, 200) |
|  | Activation ('elu') | (None, 1, 52, 200) |
|  | MaxPooling2D | (None, 1, 26, 200) |
|  | Dropout | (None, 1, 26, 200) |
| Binary classifier | Flatten | (None, 5200) |
|  | Dense | (None, 2) |
|  | Activation ('softmax') | (None, 2) |

Table S3. EEGNet

| Description | Layers | Shape |
| --- | --- | --- |
| Temporal filter | InputLayer | (None, 56, 480, 1) |
|  | Conv2D | (None, 56, 468, 40) |
| Spatial filter  (Convolution block) | Conv2D | (None, 1, 468, 40) |
|  | BatchNormalization | (None, 1, 468, 40) |
|  | Activation ('elu') | (None, 1, 468, 40) |
|  | AveragePooling2D | (None, 1, 62, 40) |
|  | Activation ('elu') | (None, 1, 62, 40) |
|  | Dropout | (None, 1, 62, 40) |
| Binary classifier | Flatten | (None, 2480) |
|  | Dense | (None, 2) |
|  | Activation ('softmax') | (None, 2) |
